# The microbial changes in subgingival plaques of orthodontic patients: a systematic review and meta-analysis of clinical trials

**DOI:** 10.1186/s12903-017-0378-1

**Published:** 2017-06-02

**Authors:** Runzhi Guo, Yifan Lin, Yunfei Zheng, Weiran Li

**Affiliations:** Department of Orthodontics, Peking University School and Hospital of Stomatology, Beijing, 100081 People’s Republic of China

**Keywords:** Periodontopathogens, Orthodontic appliance, Periodontal disease, Systematic review

## Abstract

**Background:**

Orthodontic treatment was found to have an impact on the quantity and constitution of subgingival microbiota. However, contradictory findings regarding the effects of fixed appliances on microbial changes were reported. The aim of this systematic review was to investigate the microbial changes in subgingival plaques of orthodontic patients.

**Methods:**

The PubMed, Cochrane Library, and EMBASE databases were searched up to November 20, 2016. Longitudinal studies observing microbial changes in subgingival plaques at different time points of orthodontic treatment are included. The methodological quality of the included studies was assessed by Methodological index for non-randomized studies (MINORS). The studies that reported the frequency of subgingival periodontopathogens were used for quantitative analysis. Other studies were analysed qualitatively to describe the microbial changes during orthodontic treatment.

**Results:**

Thirteen studies were selected, including two controlled clinical trials, three cohort studies and eight self-controlled studies. Four periodontopathogens, including *Aggregatibacter actinomycetemcomitans* (*Aa*), *Porphyromonas gingivalis* (*Pg*), *Prevotella intermedia* (*Pi*) and *Tannerella forsythia* (*Tf*), were analysed. Following orthodontic appliance placement, the frequencies of *Pg* and *Aa* showed no significant change (*P* = 0.97 and *P* = 0.77), whereas the frequency of *Tf* significantly increased (*P <* 0.01) during short-term observation (0–3 months). The frequency of *Pi* showed a tooth-specific difference, as it presented no significant difference (*P* = 0.25) at the site of the first molar but was significantly increased (*P* = 0.01) at the incisor. During long-term observation (> = 6 months), two studies reported that the levels of subgingival periodontopathogens exhibited a transient increase but decreased to the pretreatment levels afterwards. After removal of the orthodontic appliance, the four periodontopathogens showed no significant difference compared with before removal.

**Conclusion:**

The levels of subgingival pathogens presented temporary increases after orthodontic appliance placement, and appeared to return to pretreatment levels several months later. This indicates that orthodontic treatment might not permanently induce periodontal disease by affecting the level of subgingival periodontal pathogen levels. Further studies of high methodological quality are required to provide more reliable evidence regarding this issue.

## Background

Fixed orthodontic treatment, which is a common method for correcting malocclusion, has a close correlation with periodontal health. There are debates on the effect that applying fixed orthodontic treatment has on periodontal health. Because aligned teeth can be easily cleaned and traumatic occlusion can be relieved through orthodontic treatment, orthodontic treatment benefits periodontal conditions in the long term [1–3]. Nevertheless, plaque accumulation and gingival inflammation, including bleeding, swelling, and hyperplasia, are common during orthodontic treatment [4]. Therefore, it is likely that a fixed appliance could increase the risk of gingivitis, or even periodontitis during orthodontic treatment. The aetiology of gingivitis and periodontitis is microbial infection, resulting in an imbalance between the host and the microorganism and a change in the subgingival microorganism [5]. Fixed appliances can change the subgingival microbial environment by increasing plaque accumulation and deepening gingival sulcus [6, 7]. Some studies have reported microbial changes in the subgingival plaques of orthodontic patients, and found that the content of periodontopathogens in the subgingival plaques was significantly altered [4, 6–8]. Orthodontic appliances generally increased the level of periodontopathogens in subgingival plaques [9–12], even though Speer et al. [13] reported that the level of periodontopathogens decreased during the orthodontic treatment due to metal corrosion, which imposed toxic effects on the microorganism. However, the results are inconsistent in the scientific literature regarding a certain periodontopathogen. The aim of this systematic review was to investigate the changes in periodontopathogens throughout orthodontic treatment and to evaluate the clinical significance of these changes, such as whether and when additional periodontal treatments are needed during orthodontic treatment.

## Methods

This systematic review and meta-analysis was performed in accordance with the guidelines of the Preferred Reporting Items for Systematic Reviews and Meta-Analyses (PRISMA) checklist. There was no registration for this systematic review and meta-analysis.

### Criteria for considering studies for this review

#### Type of study

Longitudinal studies that observed the microbial changes at different time points of treatment (before, during and after treatment) were included. Randomized controlled trials (RCTs), controlled clinical trials, cohort studies and self-controlled studies were also included. Cross-sectional studies were excluded because they could not reflect the dynamic microbiological changes in orthodontic patients.

#### Type of participants

We included studies of orthodontic patients with no restrictions in terms of the characteristics of occlusion or age. Patients with periodontitis were excluded. Before appliance placement, patients with a periodontal probing depth of less than 4 mm and no periodontal attachment loss were included. Professional oral hygiene instruction was provided for all subjects. Participants with poor oral hygiene, the ones who used antibiotics or hormones 1 month before joining the study, pregnant patients and those with systematic diseases were excluded.

#### Type of intervention

For orthodontic treatment, patients with metal brackets and bands were included. Patients with ceramic bracket or lingual brackets were excluded. During treatment, mouthwash was not allowed for participants. Orthognathic surgery patients were also excluded because the surgery might disturb subgingival microorganisms.

#### Type of outcome measures

The subgingival plaque was collected by inserting a sterile dental curette or sterile paper points into the bottom of the gingival crevice [14]. The primary outcome measure was the frequency of periodontopathogen in the subgingival plaques, which referred to the percentage of the patients or teeth positive for periodontopathogens. Other microbial outcomes that reflected the microbial changes during orthodontic treatment were also included for qualitative analysis but not for quantitative analysis. Each study evaluated at least one periodontal pathogen.

### Search strategy for the identification of studies

The PubMed, Cochrane Library, and Embase databases were searched up to November 2016 with no language restrictions. The search strategy applied to PubMed is further described in Table 1. Other databases used revised search strategies with the assistance of a librarian. Furthermore, three major orthodontic journals (*American Journal of Orthodontics and Dentofacial Orthopedics, Angle Orthodontist and European Journal of Orthodontics*) from January 1991 to November 2016 and the reference lists of the selected articles were also searched.

**Table 1:** Search strategy for PubMed

Literature search was conducted up to 11/2016		PubMed results
# 1	orthodontic* OR “fixed appliance*”	61454
# 2	subgingival	5292
# 3	bacteria[Mesh] OR bacteria OR periodontopathogen* OR pathogen* OR microorganism OR microbe OR plaque OR biofilm OR microflora	2531627
#4	#1 AND #2 AND #3	89

### Selection of studies

The studies were screened, selected, and evaluated by two independent authors (Guo and Lin). Titles and abstracts were examined, and duplicate studies were eliminated. Full texts were obtained when the abstracts did not present enough information. Disagreements were resolved by discussion and consultation with a third author (Li).

### Data extraction

Study characteristics, including the study design, participants, sample size, sample site, collection time, collection method, analysis method, tested periodontopathogens and microbial outcomes, were independently extracted by two authors (Guo and Lin). It was carefully recorded whether the sample was collected from a bonded or a banded tooth, pooled or individually sampled. We contacted authors for further information when there was any absent or ambiguous information.

### Methodological quality assessment

The quality of the included studies was assessed according to the Methodological index for non-randomized studies (MINORS) by two authors (Guo and Zheng). The self-controlled studies were assessed by the first eight items of MINORS, whereas cohort studies and controlled clinical trials were assessed using all twelve items. Any disagreement was resolved by discussion with a third author (Li).

### Data synthesis

Clinical heterogeneity was gauged by assessing the characteristics of the study design, participants, sample site, the sample collection methods, the sample analysis methods and outcome measures. A meta-analysis was performed using Review Manger 5.3 (Copenhagen: The Nordic Cochrane Centre, The Cochrane Collaboration, 2011) to investigate the change in microbial frequency before and after placement and removal of the orthodontic appliance. Statistical heterogeneity was assessed by the Chi-square test and I-square index. When *I* ^2^ was between 0 and 50%, the heterogeneity was defined as relatively low. While *I* ^2^ was above 50%, the heterogeneity was defined as relatively high. Random-effects meta-analysis was performed when P ≤ 0.10 and *I* ^2^ ≥ 50%, otherwise fixed-effects meta-analysis was performed. *P*-values ≤ 0.01 were considered statistically significant.

## Results

### Search results

**Fig. 1:**
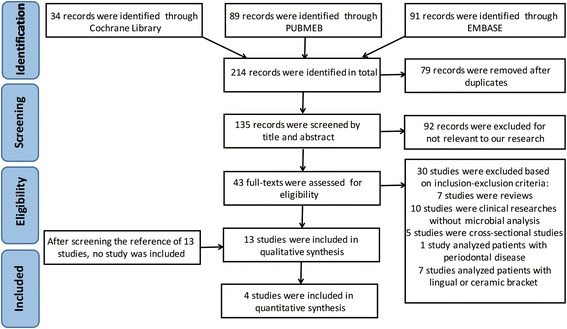
PRISMA flow diagram

A total of 214 studies were obtained from PubMed, Embase and Cochrane Library. After reviewing titles and abstracts, 43 studies proved to be potentially eligible for full-text evaluation. Thirteen studies met the eligibility criteria and were selected. Furthermore, a manual search was performed to screen the references of these 13 studies, but no studies met the criteria. Among these 13 studies, 4 studies were analysed quantitatively. The flowchart of the literature search is presented in Fig. 1.

### Assessment of methodological quality

The MINORS scores of the cohort studies and controlled clinical trials ranged from 14 to 18, out of a possible score of 24. For the self-controlled studies, the MINORS scores ranged from 9 to 13, out of a possible score of 16 (Table 2). Although there were no clear and consistent inclusion criteria for the included studies, they were identified as moderate scientific evidence considering their prospective properties and the consecutive inclusion of participants.

**Table 2:** Methodological index for non-randomized studies (MINORS)

Minors score														
Author	Year	1	2	3	4	5	6	7	8	9	10	11	12	Total
Martha.et al.	2016	2	2	2	2	2	1	2	0	0	2	1	1	17
Guo.et al.	2016	2	1	2	1	1	1	2	0	0	2	1	1	14
Yáñez-Vico.et al.	2015	2	2	2	1	1	0	2	0	1	2	0	1	14
Amezquita*.*et al.	2006	2	1	2	2	0	1	2	0	1	1	1	1	14
Choi*.*et al.	2009	2	1	2	2	1	1	2	0	1	1	1	1	15
Zivkovic Sandic*.*et al.	2014	2	1	2	2	1	1	2	0					11
Kim*.*et al.	2012	2	2	2	2	1	1	2	0					12
Liu.et al.	2011	2	2	2	2	1	1	2	0					12
LoBue*.*et al.	2008	2	1	2	1	0	1	2	0					9
Paolantonio.et al.	1999	2	1	2	2	0	2	2	0					11
Thornberg.et al.	2009	2	2	2	1	2	2	2	0					13
Sallum*.*et al.	2004	2	1	2	1	1	0	2	0					9
Ristic*.*et al.	2008	2	1	2	2	0	1	2	0					10

The items 1–12 represent: 1, a clearly stated aim; 2, inclusion of consecutive patients; 3, prospective collection of data; 4, endpoints appropriate to the aim of the study; 5, unbiased assessment of the study endpoint; 6, follow-up period appropriate to the aim of the study; 7, loss to follow-up less than 5%; 8, prospective calculation of the study size; 9, an adequate control group; 10, contemporary groups; 11, baseline equivalence of groups; and 12, adequate statistical analysis. The item scored 0 means not mentioned, 1 means reported but inadequate, and 2 means reported and adequate. The total score is 24 for cohort study and clinical controlled trial, 16 for self-controlled study

### Characteristics of the studies

The characteristics of the studies are detailed in Table 3.

**Table 3:** Characteristics of the included studies

Author/ Year	Study design	Sample size	Average age	Samplesite	Orthodontic appliance (bracket or band)	Sample collection time	Collection method	Microbial analysis method	Periodontal pathogens analyzed	Analysis based on subject or tooth	Microbiological outcome
Martha *et al.* 2016	Controlled Clinical Trials	Group A 15 Group B 10	Group A 14.4±2.45 y Group B 15.7±1.87 y	16/26/36/46	Group A Band Group B Bracket	Before appliance placement After 4–7 weeks	Sterile paper points	PCR	*Aa/Pg/Pi/Tf/* *Td/Fn/Cr/E* *c/En/Pm/C.s* *p*	Subject	Frequency of periodontopathogens in subgingival plaques
Guo *et* *al.* 2016	Controlled Clinical Trials	Group A (adults) 46 Group B (children) 62	Group A 18–32y Group B 8–15y	35/34/31/41/44/45	Bracket	Before appliance placement After 1 month After 3 months	Not mentioned	PCR	*Pg/Fn/Pi/Tf*	Subject	The percentage contents and detective amount of periodontal pathogens
Yáñez- Vico *et* *al.* 2015	Cohort study	Experimental group 61 Control group 61	21.3 ± 5.6y	15/14/34/11/45	Bracket	10 days before bracket removal 10 days after bracket removal	Sterile paper point	PCR	*Aa/Pg/Pi/Tf/* *Td*	Subject	Prevalence of periodontal pathogens in subgingival plaques
Amezquita *et* *al.* 2006	Cohort study	Experimental group 30 Control group 30	Experimental group 18.7y Control group 19.3 y	15/25/12/22/35/45	Bracke	Before appliance placement After 3 months	Sterile paper points	Culture methods	*Aa/Pg/Pi/Tf/* *Pn/Fs/Ec*	Subject	Frequency detection of periodontal pathogens in patients
Choi *et* *al.* 2009	Cohort study	Experimental group 30 Control group 30	Experimental group 20.0± 7.3y Control group 16.7±7.5 y	21/31/26/36	Incisor (bracket) Molar (band)	2 weeks before appliance removal 3 months after appliance removal	Sterile paper points	PCR	*Aa/Pg/Pi/Tf/* *Td/Pn/Cr/E* *c*	Tooth	Frequency of periodontopathogens in subgingival plaques
Zivkovic Sandic*et al.* 2014	Selfcontrolled study	Group A 14 Group B 19	19.7y (12–36)	11/16	Bracket	Group A: Before appliance placement After 1 month After 3 months Group B: Before appliance removal After 1 month After 3 months	Sterile paper points	PCR	*Aa/Pg/Pi/Tf*	Tooth	Frequency of periodontopathogens in subgingival plaques
Kim *et* *al.* 2012	Selfcontrolled study	30	16.7±6.5 y	21/31/26/36	Incisor (bracket) Molar (band)	Before appliance placement After 1 week After 3 months After 6 months	Sterile paper points	PCR	*Aa/Pg/Pi/Tf/* *Td/Pn/Cr/E* *c*	Tooth	Frequency of periodontopathogens in subgingival plaques
Liu *et* *al.* 2011	Selfcontrolled study	Group A 28 Group B 20	Group A 17.6±5.68y Group B 17.8±4.49 y	15/13/11/21/33/31/41/45	Bracket	Group A: Before appliance placement After 1 month After 3 months Group B: Before appliance removal After 1 month After3 months After 6 months	Sterile dental curette	PCR	*Pg*	Subject	Frequency of *Pg* in subgingival plaques
LoBue *et al.* 2008	Selfcontrolled study	10	13.1y	16/26/36/46	Not mentioned	Before appliance placement After 2 weeks After 4 weeks After 12 weeks	Sterile paper points	Culture methods	17 periodontalpathogens	Subject	Microorganism isolates from subgingival plaque sites
Paolantonio *et al.* 1999	Selfcontrolled study	24	18–22y	16/26/12/22 or 32/42/36/46	Lateral incisor (bracket) Molar (band)	Before appliance placement After 4 weeks After 8 weeks After 12 weeks 4 weeks after appliance removal	Sterile paper points	Culture methods	*Aa*	Subject	Frequency of *Aa* in subgingival plaques
Thornberg *et al. *2009	Selfcontrolled study	190	13.6y	16/11/24/36/31/44	Not mentioned	Before appliance placement After 6 months After 12 months After more than 12 months 3 months after appliance removed	Sterile paper points	DNA probe technique	*Aa/Pg/Pi/Tf/* *Td/Fn/Cr/E* *c*	Subject	Percentages of subjects with high pathogen counts
Sallum*et al.* 2004	Selfcontrolled study	10	16±1.8y	16/26/11	Not mentioned	Before appliance removal After 1 month	Sterile dental curette	PCR	*Aa/Pg/Pi/Pn* */Bf*	Subject	Number of sites positive for subgingival microorganisms
Ristic *et* *al.* 2008	Selfcontrolled study	32	12–18y	16/21/24	Incisor and premolar (bracket) Molar(band)	Before appliance placement After 1 month After 3 months After 6months	Sterile paper points	Culture methods	*Pi*	Tooth site	Frequency of *Pi* in subgingival plaques

#### Description of the studies

The 13 studies included 2 controlled clinical trials, 3 cohort studies and 8 self-controlled studies. Two studies [8, 15] analysed the microbial changes during the whole orthodontic treatment. Six studies [4, 6, 7, 16–18] reported the microbial changes before and after appliance placement. Three studies [19–21] reported the microbial changes before and after appliance removal. Two studies [22, 23] analysed the effects of both appliance placement and removal on microbial changes. For a long-term observation, three studies [6, 15, 18] reported at least a 6-month observation period.

#### Characteristics of interventions

Participants in all studies wore metal appliances. All of the included studies used brackets for anterior teeth and premolars, and used bands for molars except for two studies that used brackets for molars and three studies that did not mentioned.

#### Characteristics of outcome measures

Seven studies choose the frequency of periodontopathogens in subgingival plaques as the outcome indicator. Others reported percentage contents and detectable amounts of periodontopathogens in the subgingival plaques. Four studies analysed microbial changes based on the subjects, and nine studies did so based on the tooth. Four common periodontopathogens, *Aggregatibacter actinomycetemcomitans* (*Aa*), *Porphyromonas gingivalis* (*Pg*), *Prevotella intermedia* (*Pi*) and *Tannerella forsythia* (*Tf*), which are highly related to periodontal diseases and were mostly reported in the included studies, were selected for qualitative and quantitative analysis. Studies that reported other outcomes were analysed qualitatively to describe microbial trends during orthodontic treatment.

### Primary outcome

#### The microbial changes after orthodontic appliance placement

##### Short-term (1–3 months) microbial changes

*Aa*

**Fig. 2:**
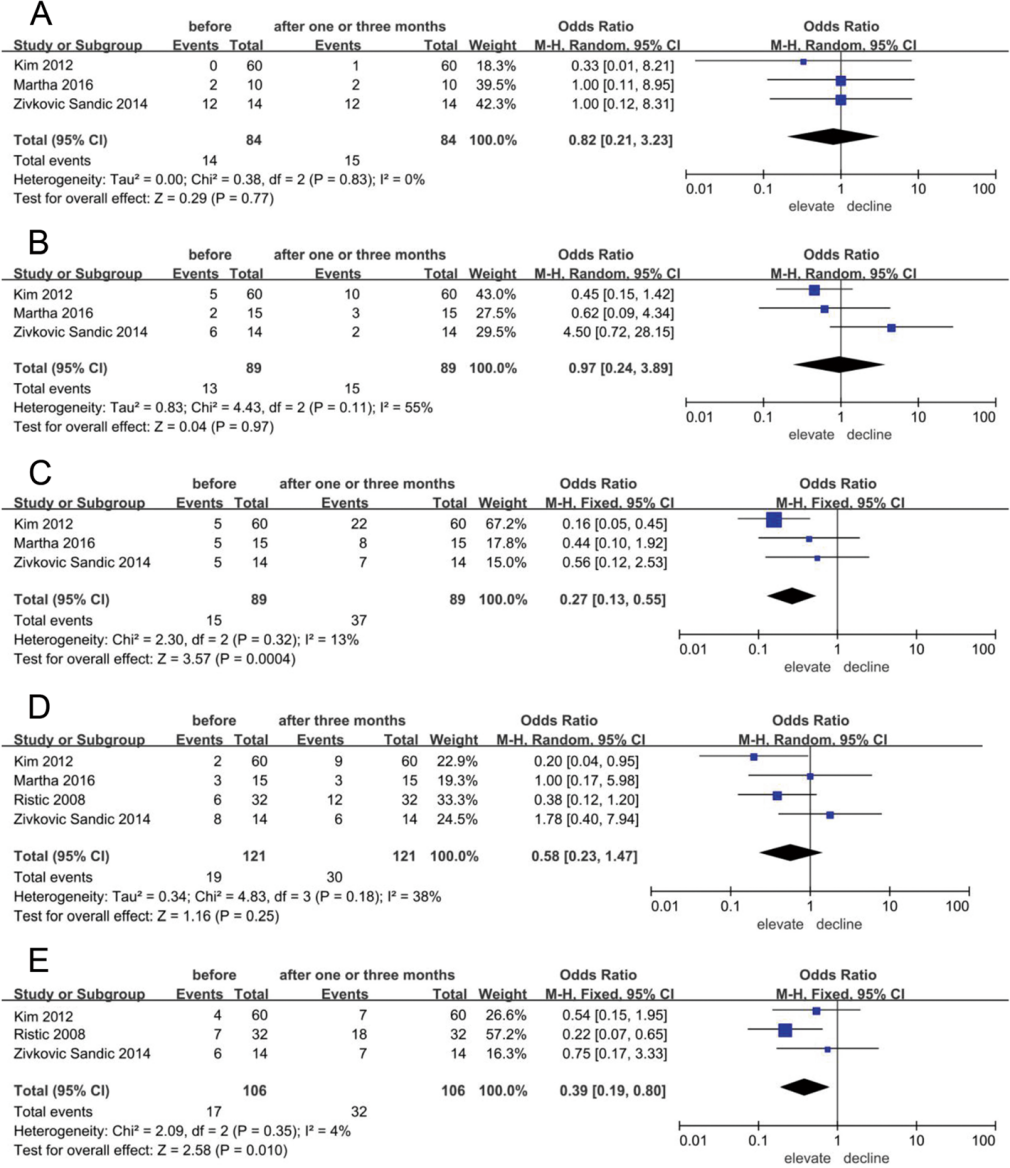
Forest plots of comparing the frequencies of four periodontopathogens before and after appliance placement. **a** The frequency of *Aa* at first molar; **b** The frequency of *Pg* at first molar; **c** The frequency of *Tf* at first molar; **d** The frequency of *Pi* at first molar; **e** The frequency of *Pi* at central incisor

Three studies [6, 16, 22] quantitatively evaluated the difference of the frequency of *Aa* in the first molar before and after appliance placement. The change was not statistically significant (odds ratio (OR), 0.82; 95% confidence interval (CI), 0.21–3.23; *P* = 0.77) according to the meta-analysis (Fig. 2a). From a qualitative perspective, six studies [6–8, 15, 16, 22] described the changes in *Aa* at the beginning of orthodontic treatment. Five of the six studies reported that the frequency of Aa was not significantly changed following the appliance placement. By contrast, Paolantonio et al. [8] found that the frequency of *Aa* in subgingival plaques significantly increased 1 month after appliance placement and maintained a high detection rate 3 months later, but decreased significantly after appliance removal.

*Pg*

A meta-analysis (Fig. 2b) from three studies [6, 16, 22] showed that the frequency of *Pg* in the first molar did not significantly change (OR, 0.97; 95% CI, 0.24–3.89; *P* = 0.97). From a qualitative perspective, six studies [4, 6, 7, 16, 22, 23] focused on *Pg* changes after orthodontic appliance placement. Among these, two studies [6, 16] found no significant change in *Pg*, but two [22, 23] showed a significant reduction in *Pg* and the remaining two [4, 7] reported a significant increase.

*Tf*

A meta-analysis (Fig. 2c) of three studies [6, 16, 22] that analysed the first molar showed that there was a significant increase (OR, 0.27; 95% CI, 0.13–0.55; *P* = 0.0004) in the frequency of *Tf* after appliance placement. From a qualitative perspective, there were five studies [4, 6, 7, 16, 22] focusing on *Tf* changes after orthodontic appliance placement. Two studies [4, 6] found a significant increase in *Tf* detection rates. The three remaining studies [7, 16, 22] found an increasing trend in *Tf* following appliance placement but without a significant difference.

*Pi*

Four studies quantitatively analysed the frequency of *Pi* at the first molar and three studies quantitatively analysed the frequency of *Pi* at the incisor. The result of meta-analysis (Fig. 2d, e) showed that there was no significant difference (OR, 0.58; 95% CI, 0.23–1.47; *P* = 0.25) in the frequency of *Pi* at the first molar after appliance placement, but that the frequency of *Pi* at the incisor did apparently increase with statistical significance (OR, 0.39; 95% CI, 0.19–0.80; *P* = 0.010). From a qualitative perspective, there were eight studies [4, 6–8, 15, 16, 18, 22] that described the changing in *Pi* after orthodontic appliance placement. Thornberg et al. [15] and Ristic et al. [18] reported the same change, in which the frequency of *Pi* increased after orthodontic appliance placement and declined several months later. Guo et al. [4] and Paolantonio et al. [8] reported a significant increase 3 months after treatment. The four remaining studies [6, 7, 16, 22] found an increasing trend, but with no statistical difference.

##### Long-term (> = 6 months) microbial changes

Three studies [6, 15, 18] monitored the changes in the microorganisms in subgingival plaques at least 6 months after orthodontic appliance placement. One of these studies monitored the whole treatment term, and the other two studies monitored 6 months after appliance placement. Two studies [15, 18] reported a transient change. One study reported that the percentage of patients with high pathogen counts increased after 6 months, and returned to pretreatment levels after 12 months. However, another study reported an increasing trend in the frequency of *Pi* after three months and a decreasing trend after six months. By contrast, Kim et al. [6] demonstrated a rising trend in the frequency of several periodontopathogens in the first six months.

#### The microbial changes after orthodontic appliance removal

*Aa*

Six studies [8, 15, 19–22] analysed *Aa* frequency after orthodontic appliance removal. Four of these studies [15, 19, 21, 22] reported that there was no significant difference. Nevertheless, Sallum et al. [20] and Paolantonio et al. [8] reported a significant reduction in the *Aa* levels.

*Pg*

Six studies [15, 19–23] analysed *Pg* changes after orthodontic appliance removal. Five of these studies found that there was no difference in the frequency of *Pg* after orthodontic appliance removal, whereas Liu et al. [23] reported a significant reduction in *Pg* levels.

*Tf*

Four studies [15, 19, 21, 22] analysed *Tf* changes after orthodontic appliance removal. Three of the four studies reported that there was no significant difference. Zivkovic Sandic et al. [22] found a significant reduction in *Tf* levels.

*Pi*

All five studies [15, 19–22] reported no apparent changes in *Pi* levels after appliance removal.

#### The diversity of microbial changes based on different teeth

Four studies [6, 18, 19, 22] analysed the changes in periodontopathogens in the subgingival plaque of a single tooth, and showed that the colonization of microorganisms on different teeth varied during orthodontic treatment. Zivkovic Sandic et al. [22] reported that only the frequency of *Tf* on the first molar showed a significant decrease after appliance removal, and that the frequency of *Tf* on incisors showed no significant difference. Other studies showed similar variability in the frequency of different microorganisms between molars and incisors.

## Discussion

The results of previous studies regarding the changes in periodontopathogens during orthodontic treatment were rather inconsistent. After the placement of orthodontic appliances, some studies reported an increasing tendency, but others reported no significant difference or a decrease in periodontopathogens. Our systematic review found that the microbial changes in subgingival plaques during orthodontic treatment might be transient. Some periodontopathogens that increased immediately after appliance placement returned to normal levels several months later.

### The factors affecting microbial changes during orthodontic treatment

There are many factors affecting the level and the content of microorganisms in subgingival plaques during orthodontic treatment, such as plaque accumulation, metal corrosion, host immunity, hormonal levels, the microbial baseline of participants and tooth movement [24–28]. Fixed appliances promote plaque accumulation, which is the critical aetiological factor of periodontal disease. Supragingival plaque accumulation influences subgingival microbial composition. Tezal et al. found that a high presence of periodontopathogens in supragingival plaques would result in the high presence of periodontopathogens in subgingival plaques [29]. In addition, orthodontic tooth movement, including intrusion and tipping, can move supragingival plaque into the subgingival sulcus, and thus affect the subgingival microorganisms. The content and virulence of bacteria are highly related to host immunity. With the equilibrium of host-microorganism, periodontopathogens can appear in the subgingival plaques of periodontally healthy subjects. Therefore, the microbial baseline of different people varies. When the content of periodontopathogens changed significantly, the disequilibrium of host-microorganism will cause periodontal inflammation. In addition, the hormonal level affects periodontal inflammation and subgingival microorganisms [30], particularly in adolescent and pregnant orthodontic patients. Metal ions, especially nickel ions, released from metal brackets and archwires could result in toxic effects on bacteria [13].

### Microbial changes after orthodontic appliance placement

#### Short-term observation (within three months)

Four main periodontopathogens were quantitatively analysed before and after the placement of an orthodontic appliance. *Pg* and *Aa* had no significant change (*P* = 0.97 and *P* = 0.77). As a member of the red complex, *Tf* was significantly increased (*P* ≤ 0.01). This result indicated that the risk of periodontal infection increased during orthodontic treatment. The change in *Pi* at the first molar showed no significant difference (*P* = 0.25), but there was a significant increase (*P* ≤ 0.01) at the incisor. Hence, considering the microbial diversity between different teeth, the result might suggest that attention should be paid especially to the teeth that are more likely to be affected by the orthodontic appliance. The iron element is necessary for the survival and reproduction of *Pi* [31]. The high iron levels may be partly responsible for the increase in *Pi* during orthodontic treatment. In addition to these four periodontopathogens, *Fusobacterium nucleatum* (*Fn*), *Prevotella nigrescens (Pn)* and *Campylobactor rectus (Cr)* also increased after appliance placement [6]. Hence, the levels of some periodontopathogens increase at the early stages of treatment.

#### Long-term observation (at least six months) of microbial changes

Generally, the long-term observation studies found a transient microbial change in that some periodontopathogens (*Pi, Tf* and *Fn*) increased at first, but then returned to the pretreatment levels several months later [15, 18]. Among these studies, Thornberg et al. [15] detected the microbial changes throughout the treatment term and found that the number of patients with high periodontopathogen counts increased six months after orthodontic appliance placement but then returned to the pretreatment level 12 months later. By contrast, Kim et al. [30] reported that the level of *Tf* remained at a high level without an obvious decrease over the first six months. This inconsistency might be due to the relatively short observation time.

### Microbial changes before and after orthodontic appliance removal

Removal of the appliance did not lead to significant changes in the frequency of the four main periodontopathogens in most of the studies, and the microbial levels were similar to those of the untreated normal controls [21]. This indicated that the microbial change was transient during orthodontic treatment, the level of subgingival periodontopathogens would return to the pretreatment level after several months. Therefore, it is reasonable that there was no significant difference in microbial changes before and after appliance removal. Only a few studies reported a decrease in the frequency of *Tf*, *Pg* and *Aa* [8, 22, 23]. These inconsistencies might be due to the different sample collection methods and microbial detection methods.

The transient increase in subgingival microorganisms can be explained by the imbalance of the host-microorganism interaction due to the orthodontic appliance and force. After several months, the host-microorganism balance was re-established, and the level of periodontopathogens returned to the pretreatment levels with improved host immunity. Although subgingival microbial levels might not be permanently affected by the orthodontic appliance, attention should also be paid to the maintenance of oral hygiene and regular periodontal examinations at the early stages of treatment when a high level of periodontopathogens is detected.

### Limitations

The main limitation of this review is the shortage of large and high-quality RCTs. The numbers of relevant research articles and patients were not sufficiently large. The observation times of the included studies were relatively short.

Moreover, clinical heterogeneity existed in individual studies. Regarding sample collection, eleven studies used sterile paper points, whereas two studies used curettes for plaque sampling. Considering the detection methods, eight studies used the PCR method to detect the 16S rRNA gene, including one study that used quantitative real-time PCR [4] and seven that used reverse transcription PCR. Another study used a DNA probe method, and four other studies used the culture method. Different teeth might have different microbial flora. However, only four of the included studies analysed their results based on the tooth, whereas nine studies pooled samples together regardless of tooth-specific differences. Hence, it is difficult to include all studies to perform a meta-analysis. Given the above factors, this review reflects only the changing trend in the subgingival microbiota. Further clinical trials with adequate methodologies and reliable analyses of the microbial changes during orthodontic treatment are needed.

In addition, we attempted to perform a meta-analysis of the microbial changes in the first molar with bands, but one of the four studies included in our meta-analysis used brackets for the first molars, which would lead to the heterogeneity in our meta-analysis.

## Conclusion

Based on our systematic review and meta-analysis, the levels of subgingival periodontopathogens temporarily increased after placement of an orthodontic appliance, and decreased thereafter or even returned to the pretreatment levels several months later. This review provides the perspective that orthodontic treatment might not permanently induce periodontal disease by affecting the level of subgingival periodontal pathogens. However, maintaining good oral hygiene and regular periodontal examinations are still top priorities for orthodontic patients, especially at the early stages of treatment. Further studies are required to assess the microbial changes throughout the orthodontic process.

## Abbreviations

*Aa*: *Aggregatibacter actinomycetemcomitan*
CI: Confidence interval
MINORS: Methodological index for non-randomized studies
OR: Odds ratio
*Pg*: *Porphyromonas gingivalis*
*Pi*: *Prevotella intermedia*
RCT: Randomized controlled trials
*Tf*: *Tannerella forsythia*
